# Supplementary material for: Genetic surveillance of Plasmodium falciparum populations following treatment policy revisions in the Greater Mekong Subregion
Source: Nat Commun. 2025 May 20;16:4689. doi: 10.1038/s41467-025-59946-1 (PMC12092748; doi:10.1038/s41467-025-59946-1)
Supplement: Supplementary file 1 — Supplementary Information [file 41467_2025_59946_MOESM1_ESM.pdf]

Genetic surveillance of *Plasmodium falciparum* populations following treatment policy revisions in the Greater Mekong Subregion

Wasakul V *et al.*

Supplementary material

Supplementary Tables 2

Supplementary Table 1. Included genetic markers with antimalarial resistance prediction. 2
Supplementary Table 2. Genotypically confirmed *P. falciparum* samples by year and province. 3
Supplementary Table 3. Distribution of *kelch13* allele variants. 4
Supplementary Table 4. Changes in PPQ-R proportions in the three largest KEL1/PLA1 clusters. 5
Supplementary Table 5. Temporal cluster distribution of *P. falciparum* samples. 6

Supplementary Figures 7

Supplementary Figure 1. Collected and genotyped *P. falciparum* (*Pf*) samples. 7
Supplementary Figure 2. Amplicon sequencing coverage of barcode loci and drug resistance loci. 8
Supplementary Figure 3. Percentage of WHO-registered *Pf* infections processed by GenRe-Mekong. 9
Supplementary Figure 4. Number of suspected cases tested in each country. 9
Supplementary Figure 5. Frequency of piperaquine-resistant parasites over three time periods. 10
Supplementary Figure 6. Relationship between PPQ resistance (PPQ-R) and *Pf* prevalence. 11
Supplementary Figure 7. Prevalence and *pm2/3* amplifications frequency changes. 12
Supplementary Figure 8. Temporal distribution of *crt* mutations and *pm23* amplifications. 13
Supplementary Figure 9. Temporal distribution of crt mutations and pm23 amplifications. 13
Supplementary Figure 10. Regional proportions of samples with *crt* mutations and *pm23* amplifications. 15
Supplementary Figure 11. Proportion of samples with combined *crt* and *pm23* genotypes, by country. 15
Supplementary Figure 12. Predicted resistance to mefloquine per province divided in three periods. 17
Supplementary Figure 13. Predicted resistance to chloroquine per province divided in three periods. 18
Supplementary Figure 14. Prevalence of KEL1/PLA1 between 2017 and 2022. 19
Supplementary Figure 15. Distribution of *mdr1* haplotypes and *kelch13* alleles. 19

Supplementary Methods 21

References 22

Supplementary Tables

Supplementary Table 1. Included genetic markers with antimalarial resistance prediction.

***P. falciparum* gene**	**Resistance classification**
kelch13[a]<br>441L, 446I, 449A, 452E, 458Y, 469Y, 469F, 476I, 479I, 481V, 493H, 515K, 522C, 527H, 537I, 537D, 538V, 539T, 543T, 553L, 561H, 568G, 574L, 575K, 579I, 580Y, 584V, 667T, 673I, 675V or 719N as homozygous call	artemisinin
***plasmepsin2/3* (*pm23*) amplification**[b]	piperaquine
WHO kelch13 mutant and multiple copies of pm23	dihydroartemisinin-piperaquine (DHA-PPQ)
crt<br>76T	chloroquine
dhfr	
108N	pyrimethamine
51I and 59R and 108N, all homozygous	sulfadoxine-pyrimethamine
dhps	
437G	sulfadoxine
dhfr* & *dhps<br>*dhfr*: 51I + 59R + 108N + *dhps*: 437G + 540E + one of *dhfr*:164L, *dhps*:581G, *dhps*:613S or *dhps*:613T with all mutants homozygous	sulfadoxine-pyrimethamine (IPTp[c])
***mdr1* amplification**[b]	mefloquine
WHO kelch13 mutant and multiple copies of mdr1	artesunate-mefloquine

Comprehensive details of genetic markers used in SPOTMalaria: https://ngs.sanger.ac.uk/production/malaria/Resource/29/20200705-GenRe-04a-SpotMalaria-0.39.pdf, pages 4-7, and phenotype prediction rules used in the GenRe-Mekong project: https://ngs.sanger.ac.uk/production/malaria/Resource/29/20200705-GenRe-05-PhenotypeRules-0.39.pdf

[a] The list of validated mutations in the BTB/POZ and the propeller domain was based on the WHO list: https://iris.who.int/handle/10665/274362
[b] Confirmatory *plasmepsin2/3* and *mdr1* amplification testing was performed using qPCR.
[c] Intermittent preventive treatment in pregnancy

Supplementary Table 2. Genotypically confirmed *P. falciparum* samples by year and province.

Country	Province	2017	2018	2019	2020	2021	2022
Cambodia	Kampong Speu				20	38	8
	Mondulkiri				4	7	7
	Pursat				6	19	84
	Ratanakiri	26					
	Stung Treng	23					
Laos	Attapeu	101	208	48	210	122	59
	Champasak	94	147	28	2	11	
	Salavan	103	99	27	15		2
	Savannakhet	284	387	212	70	116	8
	Sekong	13	9	12	9	4	
Vietnam	Binh Phuoc	51	324	24	3	4	
	Binh Thuan				7	1	
	Dak Lak	167	125	298	46		2
	Dak Nong	60	70	48	7		
	Gia Lai	272	446	523	151	73	144
	Khanh Hoa	26	48	7	1		
	Kon Tum			3			
	Ninh Thuan	43	12	13	1		2
	Phu Yen			197	55	17	9
	Quang Binh						1
	Quang Tri	28	19	7	1	1	3

Supplementary Table 3. Distribution of *kelch13* allele variants.

The proportion of samples with kelch13 variant in each country are shown as percentages for each year; all detected variants are shown. "WT" (wild type) indicates no mutation was detected in *kelch13*. "Heterozygous" indicates samples containing multiple parasites genomes carrying more than one *kelch13* mutations. "Missing" indicates parasites whose *kelch13* genotype could not be determined. "Resistant" status was determined from the list of validated alleles associated with delayed clearance, as published by WHO.[1] Full details about the classification method are is given in the SpotMalaria Technical Notes at https://www.malariagen.net/resource/29.

Country	Classification	*kelch13 allele*	2017	2018	2019	2020	2021	2022
Cambodia	Sensitive	WT	10.2%			10.0%	1.6%	7.1%
		A578S						2.0%
	Resistant	C580Y	83.7%			30.0%	54.7%	5.1%
		P553L	4.1%					
		Y493H				36.7%	29.7%	67.7%
	Undetermined	missing	2.0%			23.3%	4.7%	7.1%
		heterozygous					9.4%	11.1%
Laos	Sensitive	WT	43.2%	64.4%	30.0%	24.2%	42.7%	13.0%
	Resistant	C580Y	12.9%	22.5%	15.6%	12.7%	17.4%	49.3%
		P574L	0.8%					
		R539T	0.2%	1.2%	3.4%	45.4%	27.3%	24.6%
		Y493H	1.5%	0.1%		0.3%		
	Undetermined	missing	40.5%	10.0%	44.6%	16.0%	7.9%	13.0%
		heterozygous	0.8%	1.9%	6.4%	1.3%	4.0%	
		G357S					0.4%	
		G544R					0.4%	
Vietnam	Sensitive	WT	20.2%	11.8%	5.0%	0.7%	2.1%	2.5%
	Resistant	C469F	0.5%	0.1%				
		C580Y	54.4%	77.3%	86.0%	89.3%	95.8%	77.6%
		P553L	1.5%	0.3%				
		R539T	0.2%					
	Undetermined	missing	20.6%	8.6%	8.0%	9.6%	2.1%	18.6%
		heterozygous	2.6%	1.9%	1.0%	0.4%		1.2%

Supplementary Table 4. Changes in PPQ-R proportions in the three largest KEL1/PLA1 clusters.
The proportion of samples predicted to be resistant to piperaquine (PPQ) in the largest three KEL1/PLA1 clusters (KLV01, KLV02, KLV03) are shown, aggregated by quarter (Q1: January-March; Q2: April-June; Q3: July-September; Q4: October-December). "NA" means there are no samples from the cluster in a given period.

		PPQ-R		
		**KLV01**	**KLV02**	**KLV03**
2017	Q1	**100%**	**NA**	**NA**
	Q2	**100%**	**NA**	**NA**
	Q3	**86%**	**100%**	**NA**
	Q4	**86%**	**100%**	**NA**
2018	Q1	**96%**	**100%**	**NA**
	Q2	**94%**	**100%**	**NA**
	Q3	**100%**	**98%**	**100%**
	Q4	**96%**	**100%**	**100%**
2019	Q1	**92%**	**100%**	**100%**
	Q2	**96%**	**98%**	**90%**
	Q3	**99%**	**91%**	**91%**
	Q4	**96%**	**98%**	**87%**
2020	Q1	**94%**	**95%**	**93%**
	Q2	**82%**	**100%**	**85%**
	Q3	**20%**	**100%**	**67%**
	Q4	**17%**	**NA**	**30%**
2021	Q1	**0%**	**NA**	**25%**
	Q2	**0%**	**NA**	**8%**
	Q3	**NA**	**NA**	**0%**
	Q4	**NA**	**NA**	**0%**
2022	Q1	**NA**	**NA**	**0%**
	Q2	**NA**	**NA**	**0%**
	Q3	**NA**	**NA**	**0%**
	Q4	**NA**	**NA**	**0%**

Supplementary Table 5. Temporal cluster distribution of *P. falciparum* samples.

For each cluster, we show: the cluster label; proportion of samples per year of collection; number of sample in a cluster, proportion of samples predicted resistant to artemisinin (ART), piperaquine (PPQ), and mefloquine (MQ); chloroquine (CQ), sulfadoxine (SX) and pyrimethamine (PM); number of sample with genotyped kelch13 variant; whether the cluster is a KEL1/PLA1 haplotype. All clusters of at least 10 members are shown. Samples that could not be assigned to a cluster with these parameters were labelled as 'not clustered'.

	Proportion of samples in each year							Resistance							
Cluster	2017	2018	2019	2020	2021	2022	Sample Count	ART	PPQ	MQ	CQ	SX	PM	Kelch13	KEL1/PLA1
KLV001	5%	8%	21%	16%	4%		635	100%	89%	0%	100%	100%	100%	C580Y:622	Yes
KLV002	1%	12%	18%	4%			528	100%	98%	0%	100%	100%	100%	C580Y:524	Yes
KLV003		1%	13%	10%	16%	33%	450	100%	54%	0%	100%	100%	100%	C580Y:449	Yes
KLV004	0%	0%	0%	18%	15%	4%	202	100%	0%	13%	100%	100%	100%	R539T:190; C580Y:1	
KLV005	4%	4%	3%	3%	0%		179	100%	78%	0%	100%	100%	100%	C580Y:157	Yes
KLV006	5%	3%	1%	1%			153	6%	1%	0%	100%	0%	100%	WT:116; Y493H:6; C580Y:1; P574L:1	
KLV007	1%	1%	0%	3%	10%	9%	114	100%	1%	0%	100%	100%	100%	C580Y:108	
KLV008	5%	2%	0%				107	100%	84%	0%	100%	100%	100%	C580Y:92	Yes
KLV009	1%	1%	3%	4%			88	100%	100%	0%	100%	100%	100%	C580Y:86	Yes
KLV010	3%	2%	0%	0%			83	1%	0%	0%	100%	0%	100%	WT:68; C580Y:1	
KLV011			0%		14%		60	0%	0%	0%	0%	0%	100%	WT:59	
KLV012					1%	16%	56	100%	0%	0%	100%	100%	100%	Y493H:56	
KLV013	2%	1%	1%				53	0%	2%	0%	100%	100%	100%	WT:50	
KLV014	0%	1%	2%	1%			47	100%	85%	0%	100%	100%	100%	C580Y:46	Yes
KLV015		2%	0%	0%			44	0%	0%	0%	100%	100%	100%	WT:43	
KLV016	1%	1%			0%		41	8%	0%	0%	100%	0%	100%	WT:36; C580Y:2; P574L:1	
KLV017		1%	0%	3%			37	0%	0%	0%	100%	0%	100%	WT:36	
KLV018	2%	1%	0%				35	100%	97%	0%	100%	100%	100%	C580Y:28	Yes
KLV019	0%	1%	0%				28	0%	0%	0%	100%	0%	100%	WT:19	
KLV020		1%		0%			27	0%	0%	0%	100%	0%	100%	WT:27	
KLV021		1%	0%				26	0%	0%	0%	100%	100%	100%	WT:25	
KLV022	1%	0%	1%				26	0%	0%	0%	100%	100%	100%	WT:23	
KLV023				1%	4%	1%	25	100%	0%	0%	100%	100%	100%	C580Y:25	
KLV024	1%	1%					22	100%	100%	0%	100%	100%	100%	C580Y:19	Yes
KLV025	1%	1%	0%				22	75%	0%	0%	100%	100%	100%	C580Y:12; WT:4	
KLV026	0%		1%	1%			20	100%	94%	0%	100%	100%	100%	C580Y:20	Yes
KLV027	1%	0%	0%				20	0%	7%	0%	100%	100%	100%	WT:17	
KLV028		0%	0%	1%			19	0%	0%	0%	100%	100%	100%	WT:19	
KLV029	1%	0%	0%				19	16%	0%	0%	0%	100%	100%	WT:16; C580Y:3	
KLV030	0%	1%	0%				19	100%	0%	0%	100%	100%	100%	C580Y:15	
KLV031			0%	2%	0%		18	0%	0%	0%	100%	0%	0%	WT:16	
KLV032		0%	0%	0%	3%		18	0%	0%	0%	0%	0%	100%	WT:17	
KLV033		1%	0%	0%	0%		18	0%	0%	0%	100%	0%	100%	WT:17	
KLV034	1%	1%					18	0%	7%	0%	100%	100%	100%	WT:16	
KLV035	1%	0%					18	12%	0%	0%	100%	100%	100%	WT:7; C580Y:1	
KLV036					3%	1%	17	0%	0%	0%	100%	0%	100%	WT:17	
KLV037	1%	0%	0%				17	8%	0%	27%	0%	100%	100%	WT:11; C580Y:1	
KLV038			0%	1%	0%		16	100%	0%	0%	100%	100%	100%	R539T:15	
KLV039		1%	0%				16	100%	42%	0%	100%	100%	100%	C580Y:16	
KLV040	0%		1%	0%			16	100%	0%	0%	100%	100%	100%	C580Y:14	
KLV041	1%	0%	0%				16	0%	0%	0%	100%	0%	100%	WT:9	
KLV042	0%	1%					15	100%	0%	0%	100%	100%	100%	C580Y:14	
KLV043				2%	0%	1%	13	100%	0%	0%	100%	100%	100%	Y493H:13	
KLV044	0%	1%					13	0%	0%	0%	100%	0%	100%	WT:12	
KLV045		1%					13	0%	0%	0%	100%	0%	100%	WT:13	
KLV046	1%	0%					13	55%	0%	0%	100%	0%	100%	C580Y:6; WT:5	
KLV047		1%					12	0%	0%	0%	100%	0%	100%	WT:12	
KLV048		1%					12	0%	20%	0%	100%	0%	100%	WT:12	
KLV049	0%	0%	0%				12	100%	100%	0%	100%	100%	100%	C580Y:12	Yes
KLV050	0%	0%		0%			12	100%	0%	0%	100%	100%	100%	C580Y:10	
KLV051	0%	0%	0%	1%			12	0%	0%	0%	0%	0%	100%	WT:9	
KLV052	1%	0%					12	100%	100%	NA	100%	100%	100%	C580Y:5	Yes
KLV053	1%	0%					12	33%	11%	0%	100%	100%	100%	WT:2; C580Y:1	
KLV054	1%	0%					12	100%	0%	0%	100%	100%	100%	P553L:11	
KLV055		1%					11	0%	0%	0%	0%	0%	0%	WT:11	
KLV056	1%	0%		0%			11	22%	0%	0%	100%	100%	100%	WT:7; Y493H:2	
KLV057	0%	0%	0%	1%			11	0%	0%	0%	100%	0%	100%	WT:9	
KLV058	0%	0%					11	100%	100%	0%	100%	100%	100%	C580Y:11	Yes
KLV059	1%						11	0%	0%	NA	0%	100%	100%	WT:5	
KLV060		1%					10	0%	0%	NA	100%	0%	100%	WT:10	
KLV061		1%					10	100%	0%	0%	100%	100%	100%	C580Y:10	
KLV062		1%					10	0%	0%	0%	100%	0%	100%	WT:7	
KLV063	0%	0%	0%				10	10%	0%	0%	100%	0%	100%	WT:9; P574L:1	
KLV064	1%						10	0%	0%	NA	0%	0%	100%	WT:10	
Not clustered	55%	43%	30%	26%	26%	36%									
Total samples	1291	1894	1447	608	413	329									

Supplementary Figures

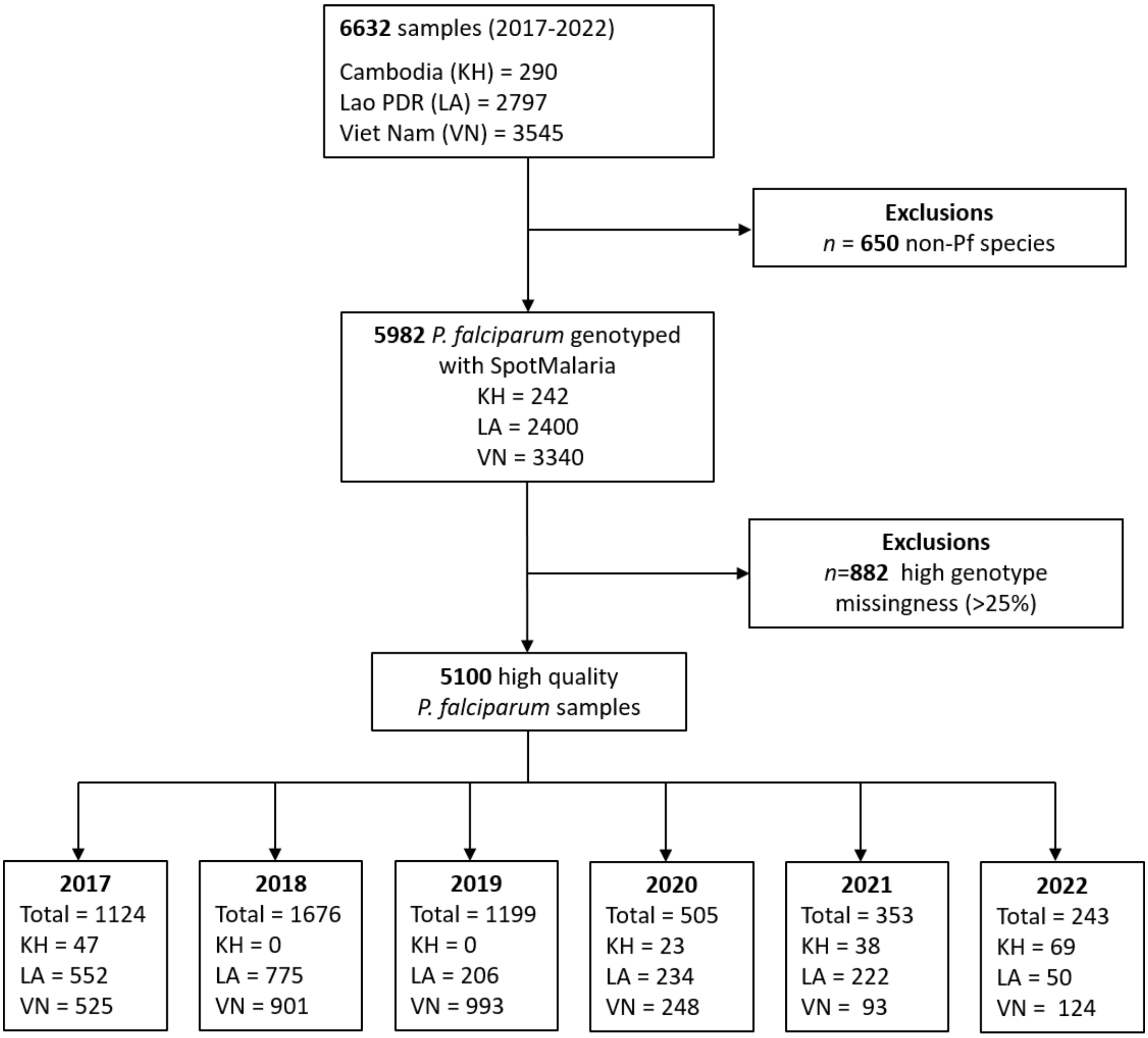

Supplementary Figure 1. Collected and genotyped *P. falciparum* (*Pf*) samples.
The diagram shows how the analysed sample set was derived from *Pf* samples collected by GenRe-Mekong, and the composition of this sample set by country and year.

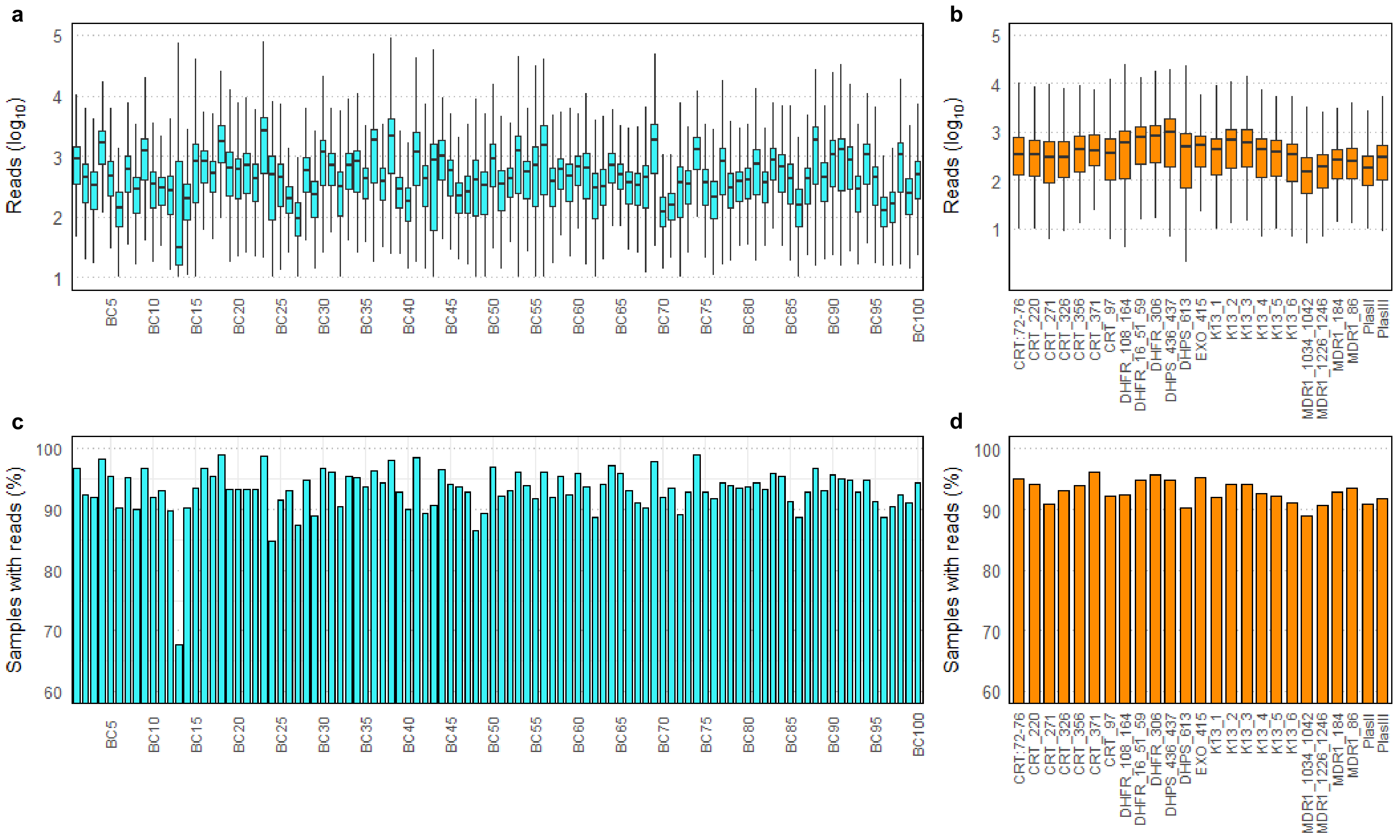

Supplementary Figure 2. Amplicon sequencing coverage of barcode loci (*n* = 100) and drug resistance loci (*n* = 25).
Boxplots show the distribution of sequencing read counts (log_{10} transformed) across individual barcodes (**a**) and drug resistance markers (**b**); center line shows the median, box limits indicate upper and lower quartiles, whiskers extend to 1.5x interquartile range. Barcharts show the proportions of samples with ≥10 reads at each barcode locus (**c**) and each drug resistance marker (**d**).

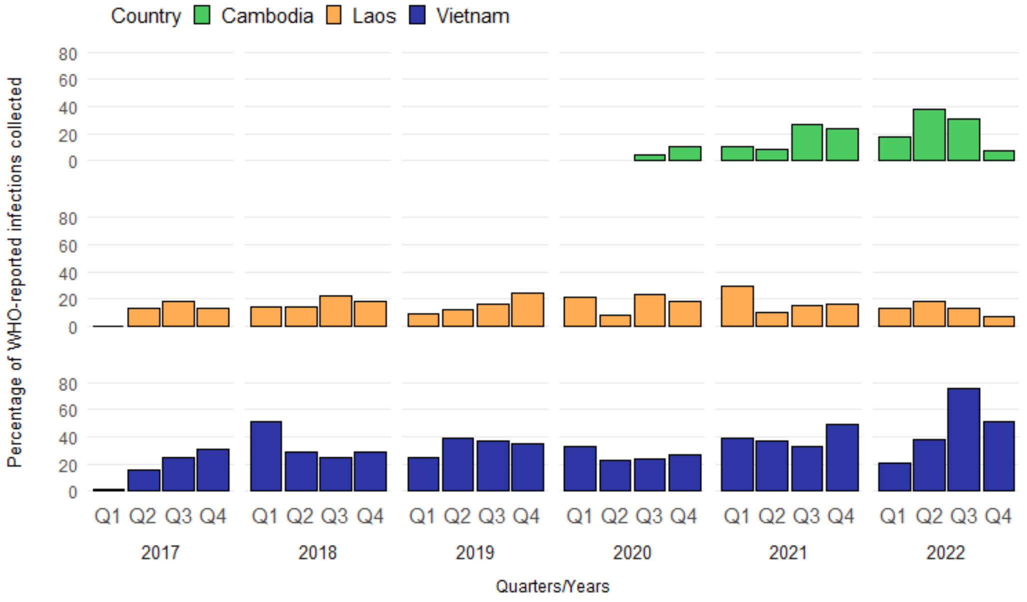

Supplementary Figure 3. Percentage of WHO-registered *Pf* infections processed by GenRe-Mekong.
Between January 2017 to December 2022, 32.7% (3,340/10,212) and 16.2% (2,400/14,811) WHO-reported *Pf* infections in Vietnam and Laos were sampled by the GenRe-Mekong project, respectively. For Cambodia, 21.0% (242/1,151) of WHO-reported *P. falciparum* infections occurred between 2020 and 2022 were sampled GenRe-Mekong.[2-4]

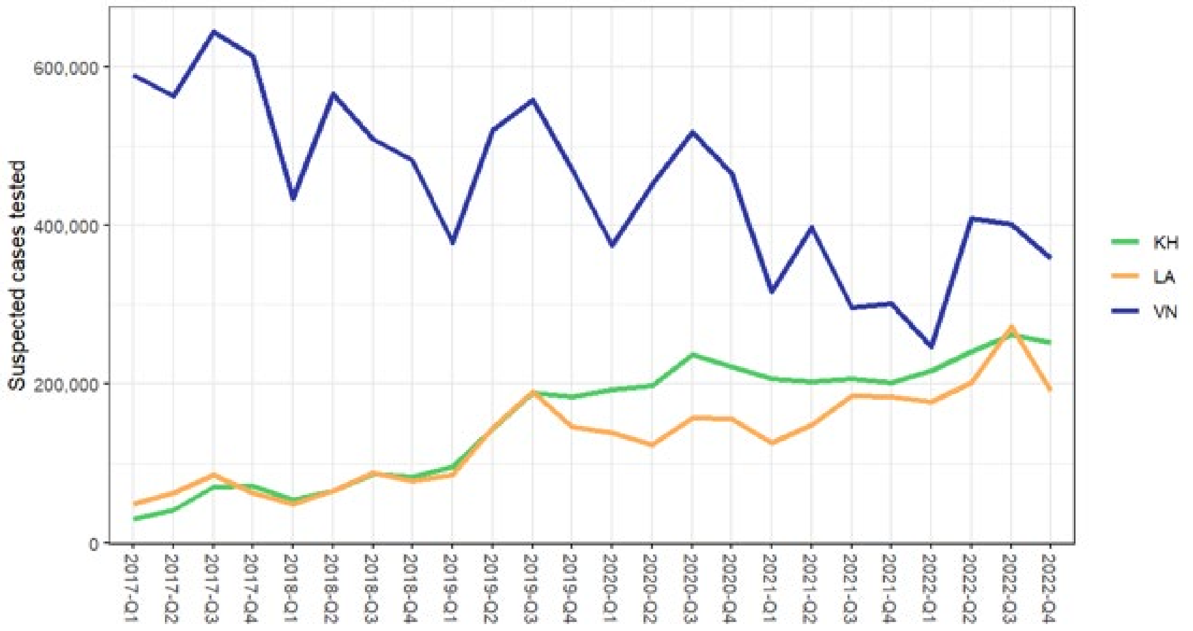

Supplementary Figure 4. Number of suspected cases tested in each country.
Numbers reported to the WHO Mekong Malaria Elimination Programme.[2-4] KH: Cambodia, LA: Lao PDR and VN: Vietnam.

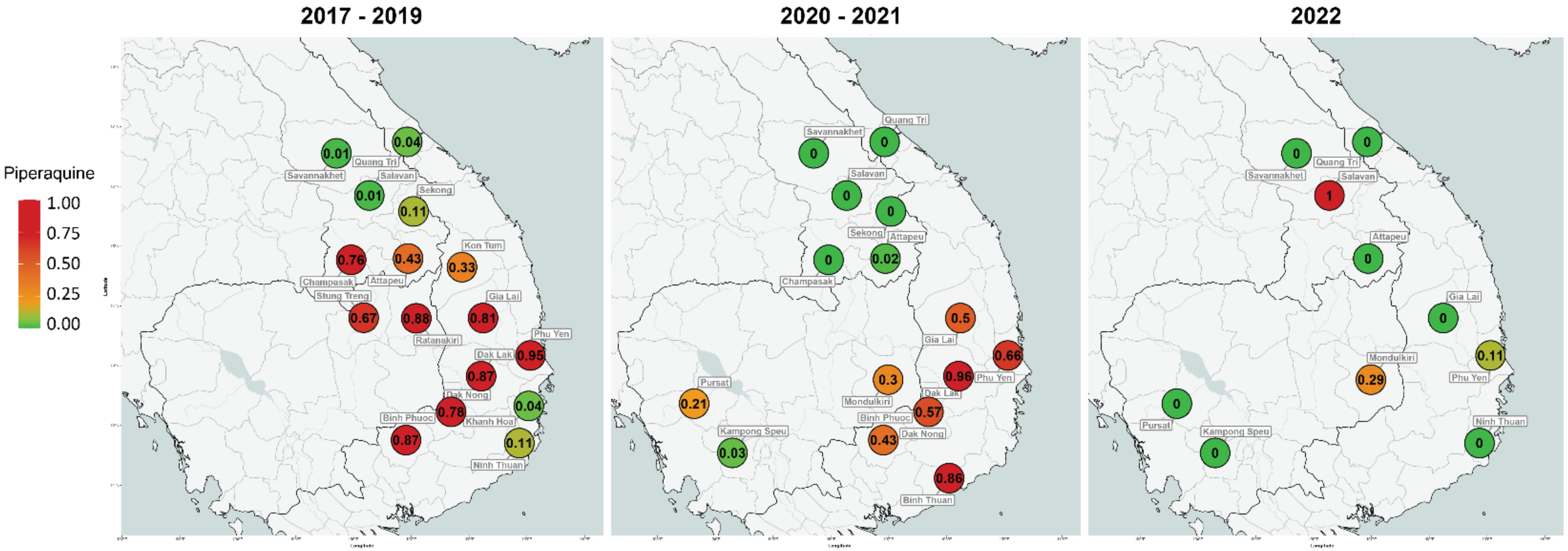

Supplementary Figure 5. Frequency of piperaquine-resistant parasites over three time periods.

The predictor marker for piperaquine resistance was the *pm23* amplifications. Left panel: 2017-2019, middle: 2020-2021, right: January-December 2022. Marker colours reflect resistance prevalence, ranging from 0 to 1, where 0 means no parasite were predicted to be resistant, and 1 means 100% of the parasites in the province carried the relevant resistance markers. A marker is shown only if there are at least 2 samples from the province (e.g.: only two samples were samples were present in Salavan in 2022). Sample number for each province are shown in Supplementary Table 2.

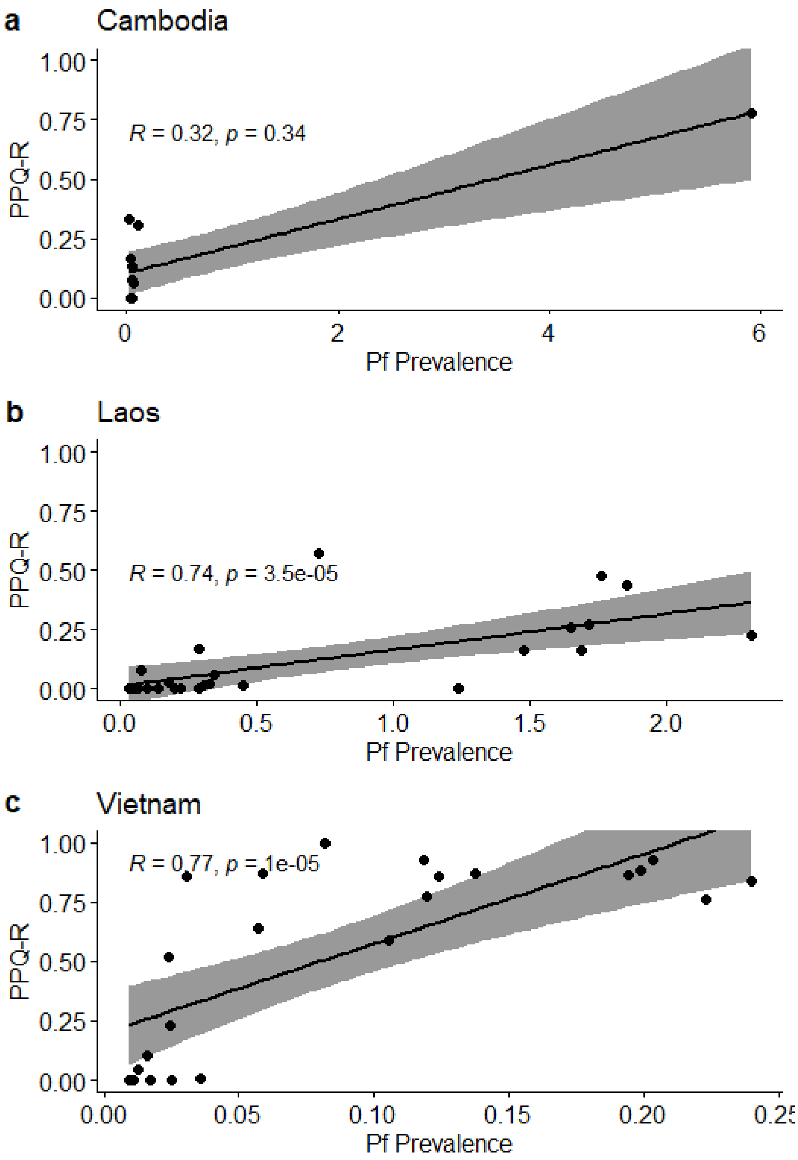

Supplementary Figure 6. Relationship between PPQ resistance (PPQ-R) and *Pf* prevalence.
For each country, we show a Spearman's rank correlation analysis highlighting the relationship between levels of PPQ resistance (PPQ-R) and prevalence of *Plasmodium falciparum* (*Pf*) across three countries: **a** Cambodia (sample size, n = 229), **b** Laos (n = 1437), and **c** Vietnam (n = 2852). Samples with missing *plasmepsin2/3* genotypes are excluded from the analysis. Data points represents quarterly observations (n = 24 per country) over six years. Each panel shows the linear regression fit (black line) with 95% confidence intervals (shaded gray area). The correlation coefficient (R) and associated p-value are shown in each panel. The analysis indicates a weak non-significant correlation in Cambodia, but strong and significant positive correlations in Laos and Vietnam. *Pf* malaria prevalence was estimated from WHO data.[2-4]

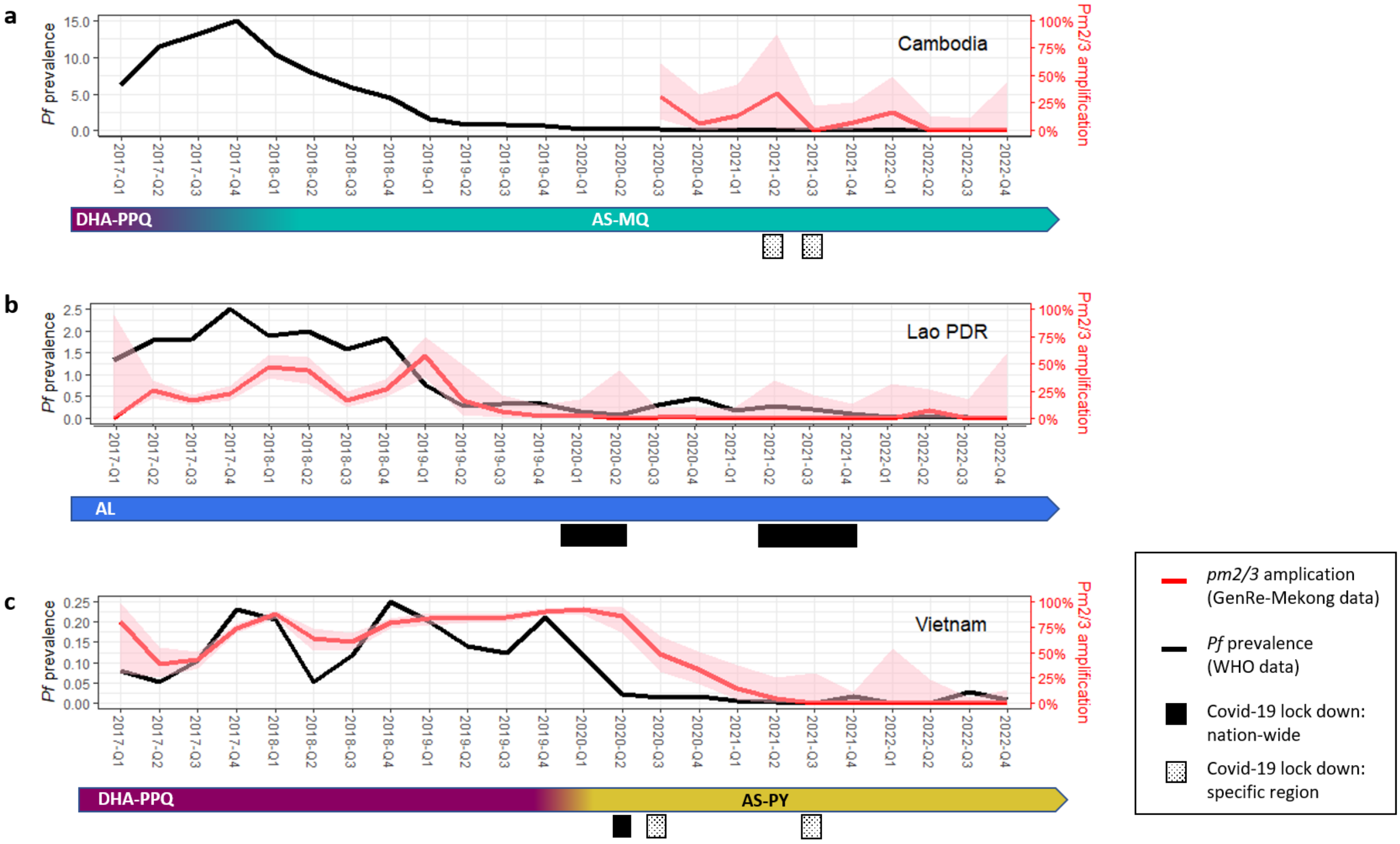

Supplementary Figure 7. Prevalence and *pm2/3* amplifications frequency changes.
Panels: (**a**) Cambodia, (**b**) Laos and (**c**) Vietnam. *Pf* malaria prevalence (black lines, left-axis) was derived from WHO data,[2-4] while *pm23* amplification frequencies (red lines, right-axis) were derived from GenRe-Mekong genotypes. Shaded areas represent 95% confidence intervals, with wider intervals reflecting smaller sample sizes. The *Pf* prevalence scale varies between countries. First line ACT treatment for uncomplicated malaria, according to national policy, and COVID-19 lockdown restrictions are shown under each graph. Cambodia switched ACT from dihydroartemisinin-piperaquine (DHA-PPQ) to artesunate-mefloquine (AS-MQ) starting 2017,[5,6] and did not implement a nationwide lockdown but focused on localized restrictions: in Phnom Penh and Ta Khmau in April-May 2021, and provinces bordering Thailand in July-August 2021.[7] GenRe-Mekong started routine surveillance in Cambodia in Q3 2020. Laos continued the use of artemether-lumefantrine (AL) throughout the study period, and lockdowns were imposed nationally March-May 2020[8] and April-December 2021.[9] Vietnam changed frontline ACT from DHA-PPQ to artesunate-pyronaridine (AS-PYR) in five provinces in late Q1 2020,[10,11] and COVID-19 lockdowns were imposed nationwide in April-May 2020; in Danang July-August 2020; and in Hanoi and the Southern region in July-August 2021.[12,13]

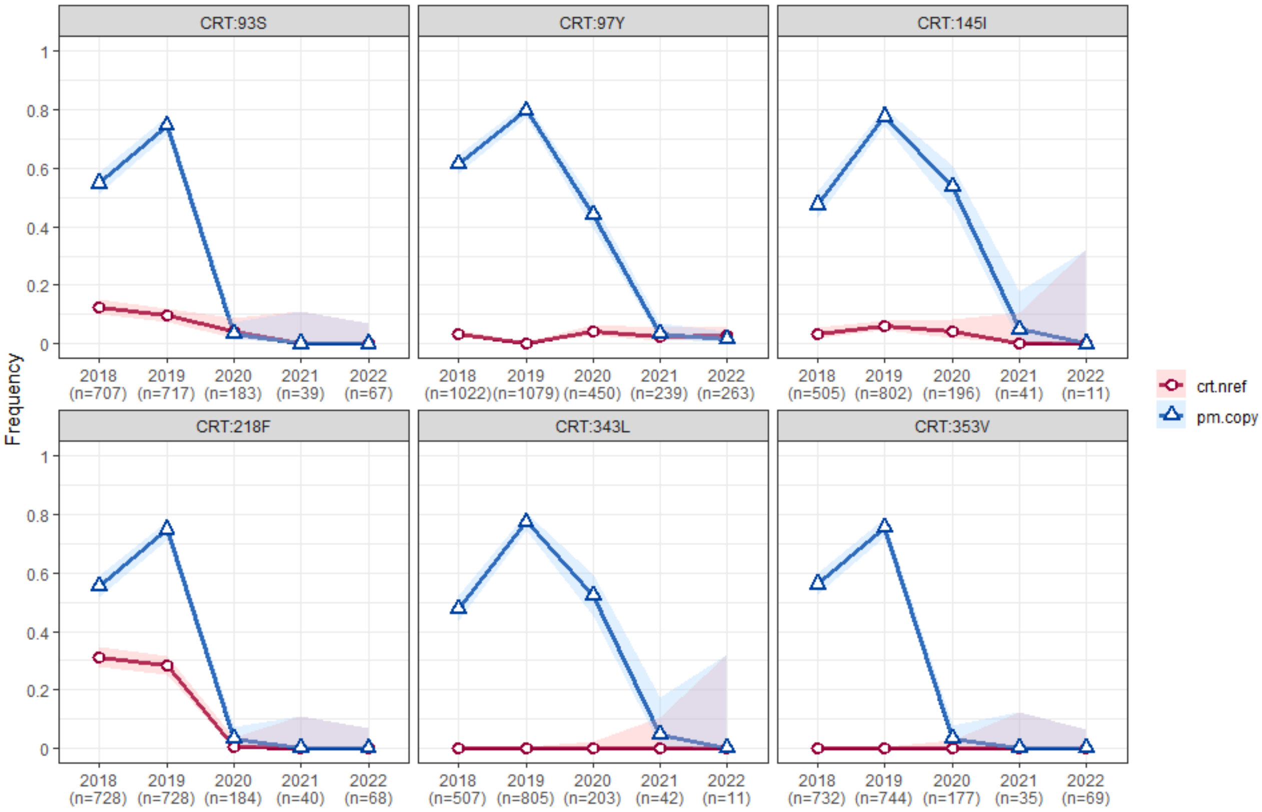

Supplementary Figure 8. Temporal distribution of *crt* mutations and *pm23* amplifications.
The proportion of samples carrying *crt* mutations is shown by year in red (crt.nref), while the proportion of *pm23* amplifications is shown in blue (pm.copy), with value ranging from 0 (no samples carrying the resistant allele) to 1 (100% of samples carrying the resistant allele). Sample sizes (n) are indicated on the x-axis for each year. Shaded areas represent 95% confidence intervals, with wider intervals reflecting smaller sample sizes. Analysis includes only samples for which both the *crt* and *pm23* genotypes are available.

(Next Page)

Supplementary Figure 9. Temporal distribution of crt mutations and pm23 amplifications.
The proportion of samples carrying *crt* mutations is shown by year in red (crt.nref), while the proportion of *pm23* amplifications is shown in blue (pm.copy) for (**a**) Cambodia (KH), (**b**) Laos (LA) and (**c**) Vietnam (VN), with value ranging from 0 (no samples carrying the resistant allele) to 1 (100% of samples carrying the resistant allele). Sample sizes (n) are indicated on the x-axis for each year. Shaded areas represent 95% confidence intervals, with wider intervals reflecting smaller sample sizes. Analysis includes only samples for which both the *crt* and *pm23* genotypes are available.

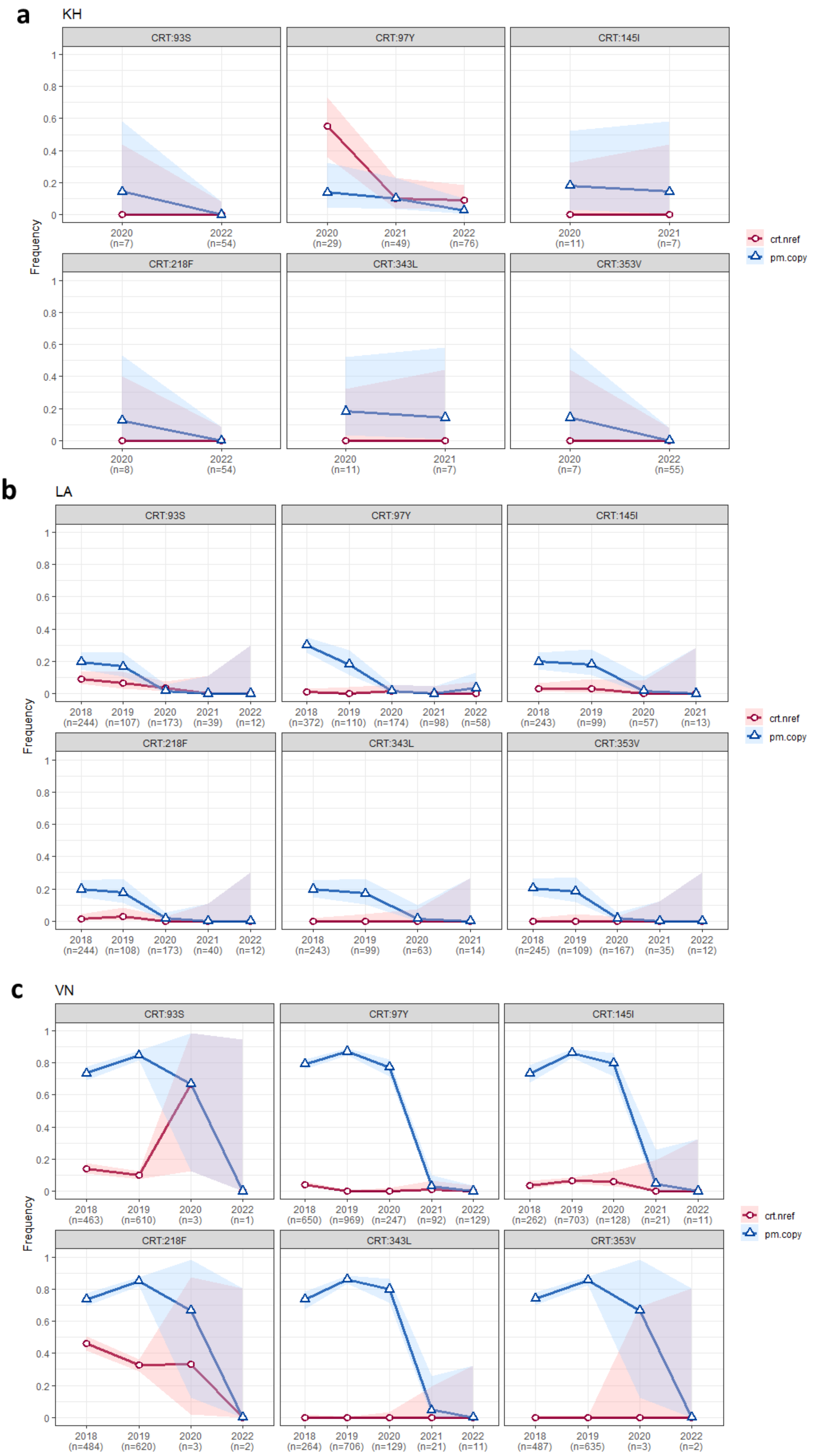

a
KH
CRT:93S
2020 (n=7)
2022 (n=54)
CRT:97Y
2020 (n=29)
2021 (n=49)
2022 (n=76)
CRT:145I
2020 (n=11)
2021 (n=7)
CRT:218F
2020 (n=8)
2022 (n=54)
CRT:343L
2020 (n=11)
2021 (n=7)
CRT:353V
2020 (n=7)
2022 (n=55)
Frequency
0
0.2
0.4
0.6
0.8
1
crt.nref
pm.copy
b
LA
CRT:93S
2018 (n=244)
2019 (n=107)
2020 (n=173)
2021 (n=39)
2022 (n=12)
CRT:97Y
2018 (n=372)
2019 (n=110)
2020 (n=174)
2021 (n=98)
2022 (n=58)
CRT:145I
2018 (n=243)
2019 (n=99)
2020 (n=57)
2021 (n=13)
CRT:218F
2018 (n=244)
2019 (n=108)
2020 (n=173)
2021 (n=40)
2022 (n=12)
CRT:343L
2018 (n=243)
2019 (n=99)
2020 (n=63)
2021 (n=14)
CRT:353V
2018 (n=245)
2019 (n=109)
2020 (n=167)
2021 (n=35)
2022 (n=12)
Frequency
crt.nref
pm.copy
c
VN
CRT:93S
2018 (n=463)
2019 (n=610)
2020 (n=3)
2022 (n=1)
CRT:97Y
2018 (n=650)
2019 (n=969)
2020 (n=247)
2021 (n=92)
2022 (n=129)
CRT:145I
2018 (n=262)
2019 (n=703)
2020 (n=128)
2021 (n=21)
2022 (n=11)
CRT:218F
2018 (n=484)
2019 (n=620)
2020 (n=3)
2022 (n=2)
CRT:343L
2018 (n=264)
2019 (n=706)
2020 (n=129)
2021 (n=21)
2022 (n=11)
CRT:353V
2018 (n=487)
2019 (n=635)
2020 (n=3)
2022 (n=2)
Frequency
crt.nref
pm.copy

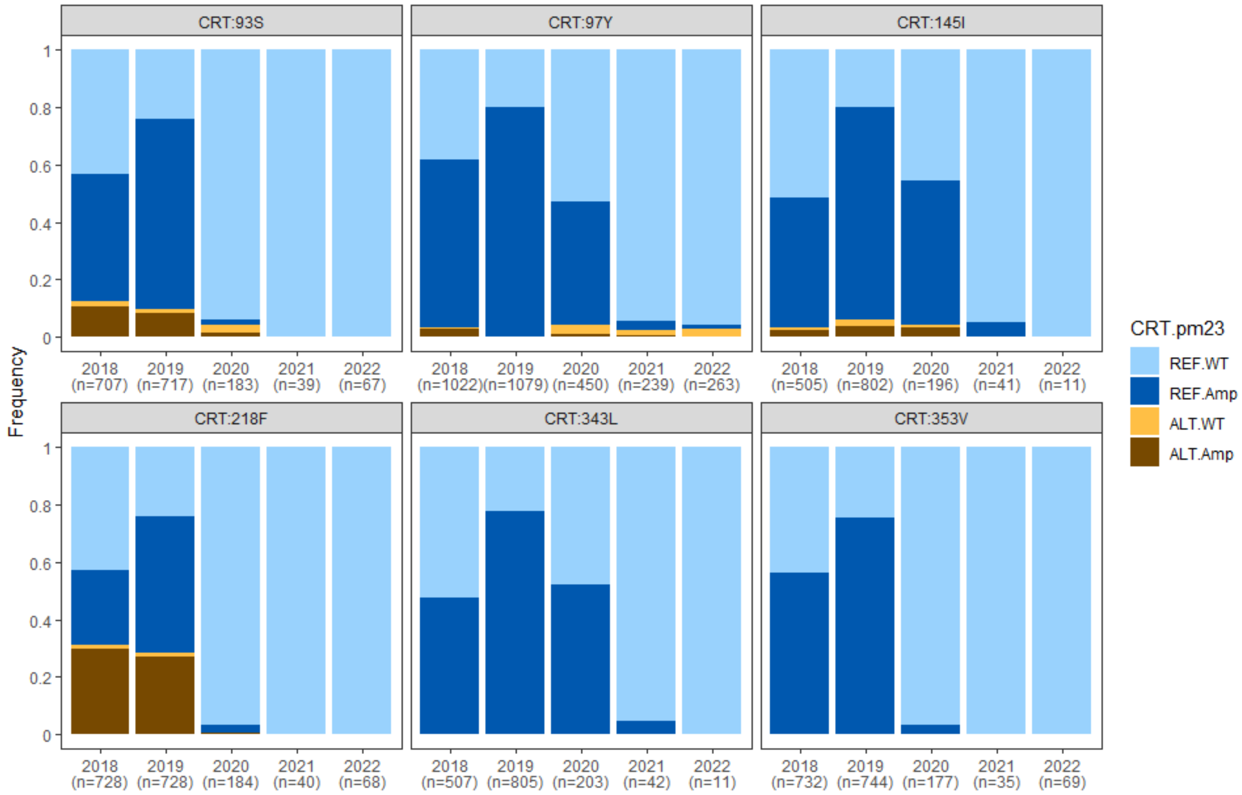

Supplementary Figure 10. Regional proportions of samples with *crt* mutations and *pm23* amplifications. The stacked bar plots show the proportion of each genotype combination in each year: wild-type for both *crt* and *pm23* (REF.WT, light blue), wild-type *crt* with *pm23* amplification (REF.Amp, navy)**,** *crt* mutant with single-copy *pm23* (ALT.WT, yellow), and *crt* mutant with *pm23* amplification (ALT.Amp, brown). Sample sizes (n) for each year are shown in parentheses below the x-axis.

(Next page)

Supplementary Figure 11. Proportion of samples with combined *crt* and *pm23* genotypes, by country. The stacked bar plots show the proportion of each genotype combination in each year for (**a**) Cambodia (KH), (**b**) Laos (LA) and (**c**) Vietnam (VN): wild-type for both *crt* and *pm23* (REF.WT, light blue), wild-type *crt* with *pm23* amplification (REF.Amp, navy)**,** *crt* mutant with single-copy *pm23* (ALT.WT, yellow), and *crt* mutant with *pm23* amplification (ALT.Amp, brown). Sample sizes (n) for each year are shown in parentheses below the x-axis.

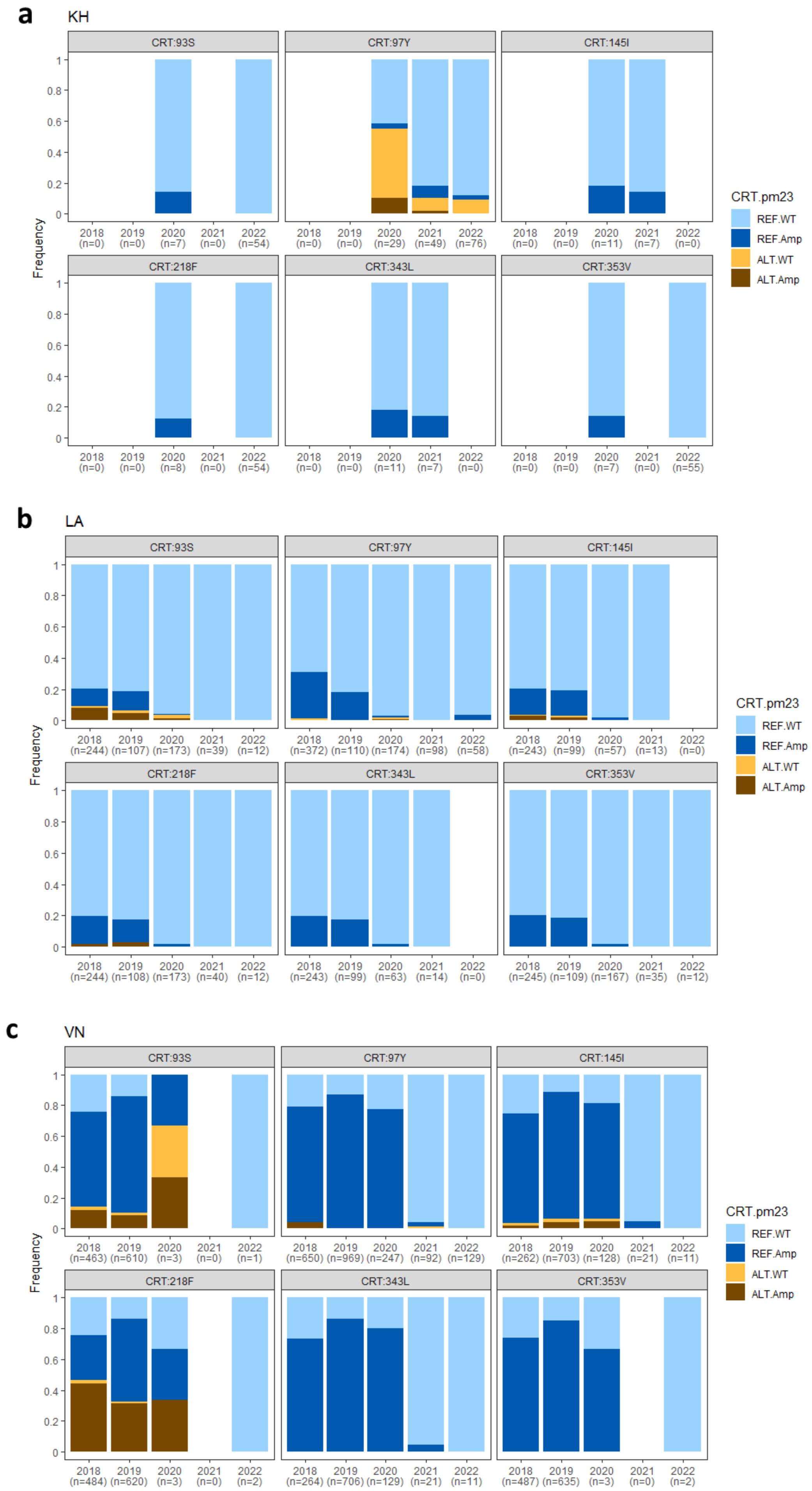
a
KH
CRT:93S
CRT:97Y
CRT:145I
CRT:218F
CRT:343L
CRT:353V
Frequency
1
0.8
0.6
0.4
0.2
0
2018 (n=0) 2019 (n=0) 2020 (n=7) 2021 (n=0) 2022 (n=54)
2018 (n=0) 2019 (n=0) 2020 (n=29) 2021 (n=49) 2022 (n=76)
2018 (n=0) 2019 (n=0) 2020 (n=11) 2021 (n=7) 2022 (n=0)
2018 (n=0) 2019 (n=0) 2020 (n=8) 2021 (n=0) 2022 (n=54)
2018 (n=0) 2019 (n=0) 2020 (n=11) 2021 (n=7) 2022 (n=0)
2018 (n=0) 2019 (n=0) 2020 (n=7) 2021 (n=0) 2022 (n=55)
CRT.pm23
REF.WT
REF.Amp
ALT.WT
ALT.Amp
b
LA
CRT:93S
CRT:97Y
CRT:145I
CRT:218F
CRT:343L
CRT:353V
Frequency
2018 (n=244) 2019 (n=107) 2020 (n=173) 2021 (n=39) 2022 (n=12)
2018 (n=372) 2019 (n=110) 2020 (n=174) 2021 (n=98) 2022 (n=58)
2018 (n=243) 2019 (n=99) 2020 (n=57) 2021 (n=13) 2022 (n=0)
2018 (n=244) 2019 (n=108) 2020 (n=173) 2021 (n=40) 2022 (n=12)
2018 (n=243) 2019 (n=99) 2020 (n=63) 2021 (n=14) 2022 (n=0)
2018 (n=245) 2019 (n=109) 2020 (n=167) 2021 (n=35) 2022 (n=12)
CRT.pm23
REF.WT
REF.Amp
ALT.WT
ALT.Amp
c
VN
CRT:93S
CRT:97Y
CRT:145I
CRT:218F
CRT:343L
CRT:353V
Frequency
2018 (n=463) 2019 (n=610) 2020 (n=3) 2021 (n=0) 2022 (n=1)
2018 (n=650) 2019 (n=969) 2020 (n=247) 2021 (n=92) 2022 (n=129)
2018 (n=262) 2019 (n=703) 2020 (n=128) 2021 (n=21) 2022 (n=11)
2018 (n=484) 2019 (n=620) 2020 (n=3) 2021 (n=0) 2022 (n=2)
2018 (n=264) 2019 (n=706) 2020 (n=129) 2021 (n=21) 2022 (n=11)
2018 (n=487) 2019 (n=635) 2020 (n=3) 2021 (n=0) 2022 (n=2)
CRT.pm23
REF.WT
REF.Amp
ALT.WT
ALT.Amp

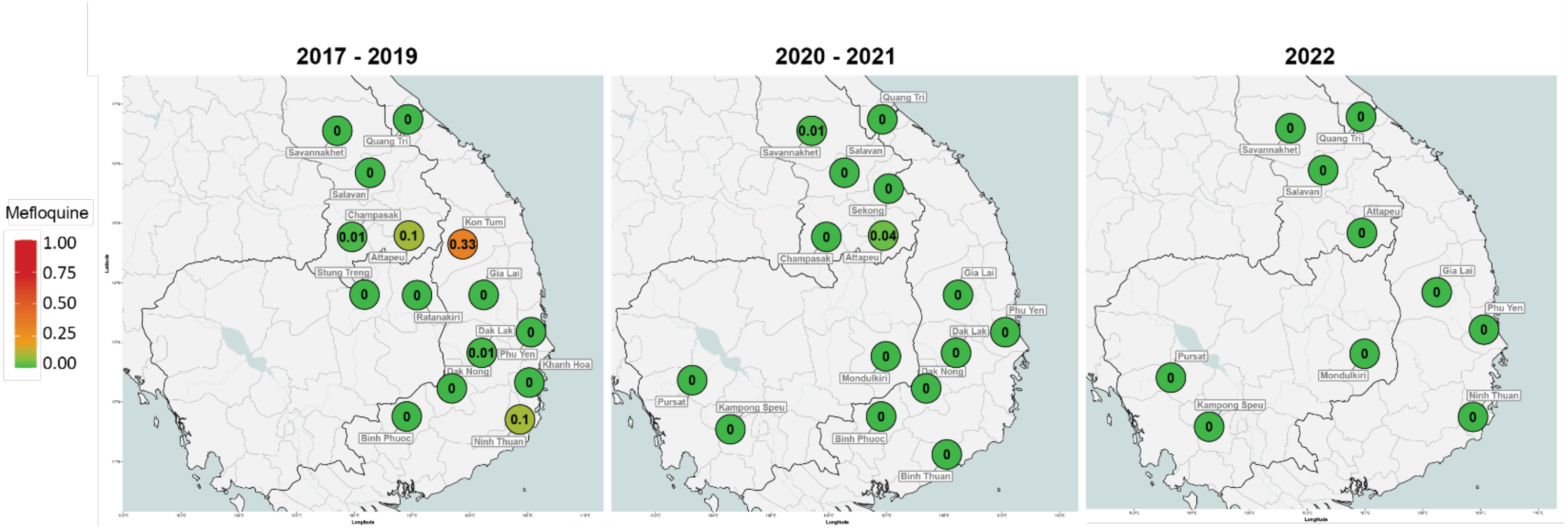

Supplementary Figure 12. Predicted resistance to mefloquine per province divided in three periods.
Left panel: 2017-2019, middle: 2020-2021, right: January-December 2022. Marker colours reflects resistance prevalence, ranging from 0 to 1, where 0 means no parasite were predicted to be resistant, and 1 means 100% of the parasites in the province carried the relevant resistance marker. A marker appears when at least 2 samples were processed from the province. Sample number for each province are shown in Supplementary Table 2.

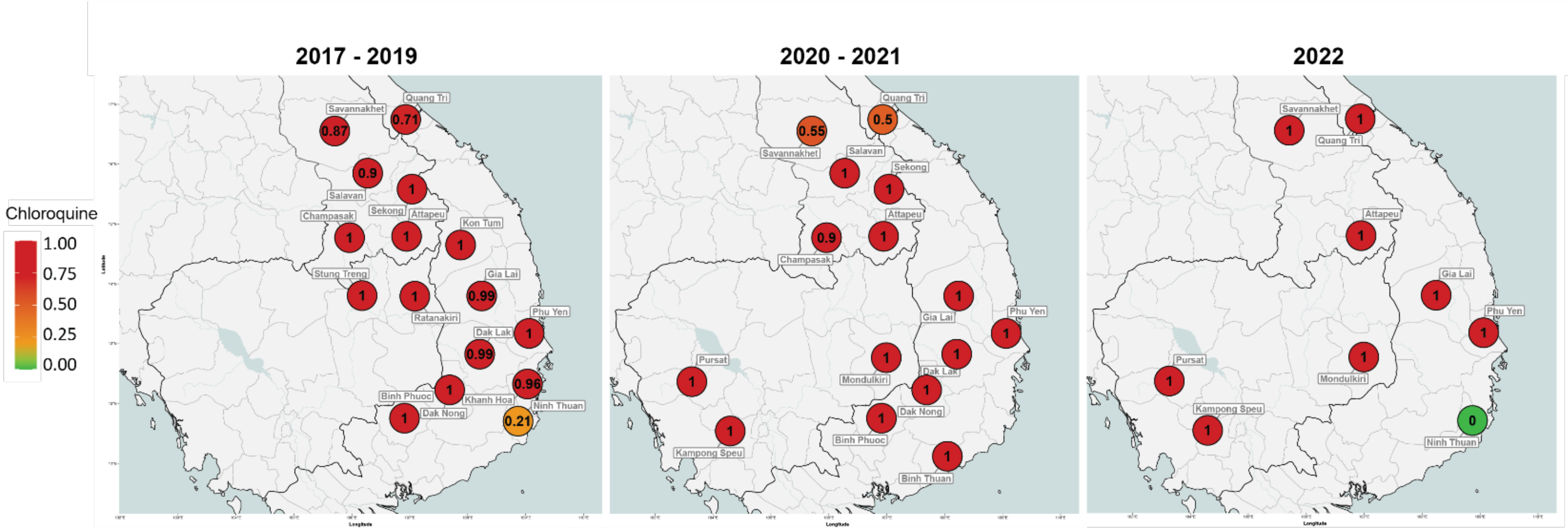

Supplementary Figure 13. Predicted resistance to chloroquine per province divided in three periods.
Left panel: 2017-2019, middle: 2020-2021, right: January-December 2022. Marker colours reflects resistance prevalence, ranging from 0 to 1, where 0 means no parasite were predicted to be resistant, and 1 means 100% of the parasites in the province carried the relevant resistance marker. A marker appears when at least 2 samples were processed from the province. Sample number for each province are shown in Supplementary Table 2.

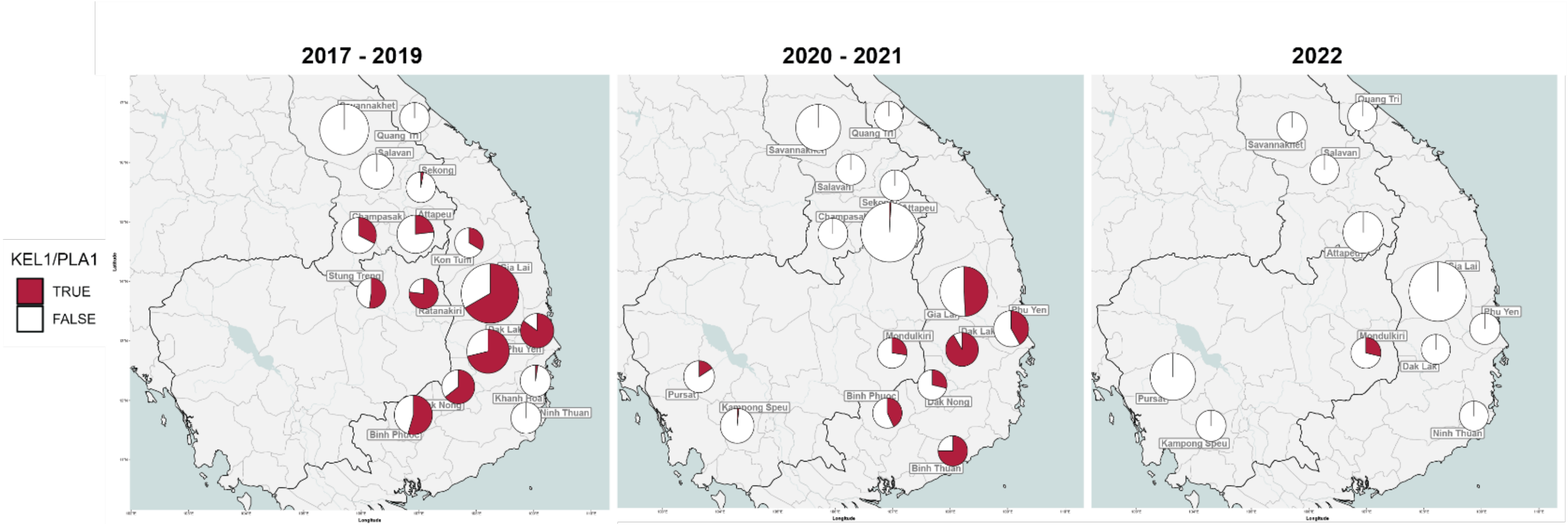

Supplementary Figure 14. Prevalence of KEL1/PLA1 between 2017 and 2022.
Pie charts show the proportions of samples classified as KEL1/PLA1, having both C580Y *kelch13* mutation and *plasmepsin 2/3* amplification, in the three periods. Left panel: 2017-2019, middle: 2020-2021, right: January-December 2022. The pie size reflects the sample number in the province. Sample number for each province are shown in Supplementary Table 2.

(Next page)

Supplementary Figure 15. Distribution of *mdr1* haplotypes and *kelch13* alleles.
Panels show the proportions of parasites carrying different *mdr1* haplotypes (a) and *kelch13* alleles (b) in three time periods (left: 2017-2019, middle: 2020-2021, right: Jan-Dec 2022. NYD is the wild-type *mdr1* haplotype. Parasites with wild-type (WT) *kelch13* alleles are predicted to be sensitive to artemisinin, while other *kelch13* alleles have been associated with delayed parasite clearance and therefore predicted to be artemisinin-resistant, with the exception of G357S and G544R which are not in the WHO validated marker list, and thus their association with artemisinin resistance is undetermined. Sample number for each province are shown in Supplementary Table 2.

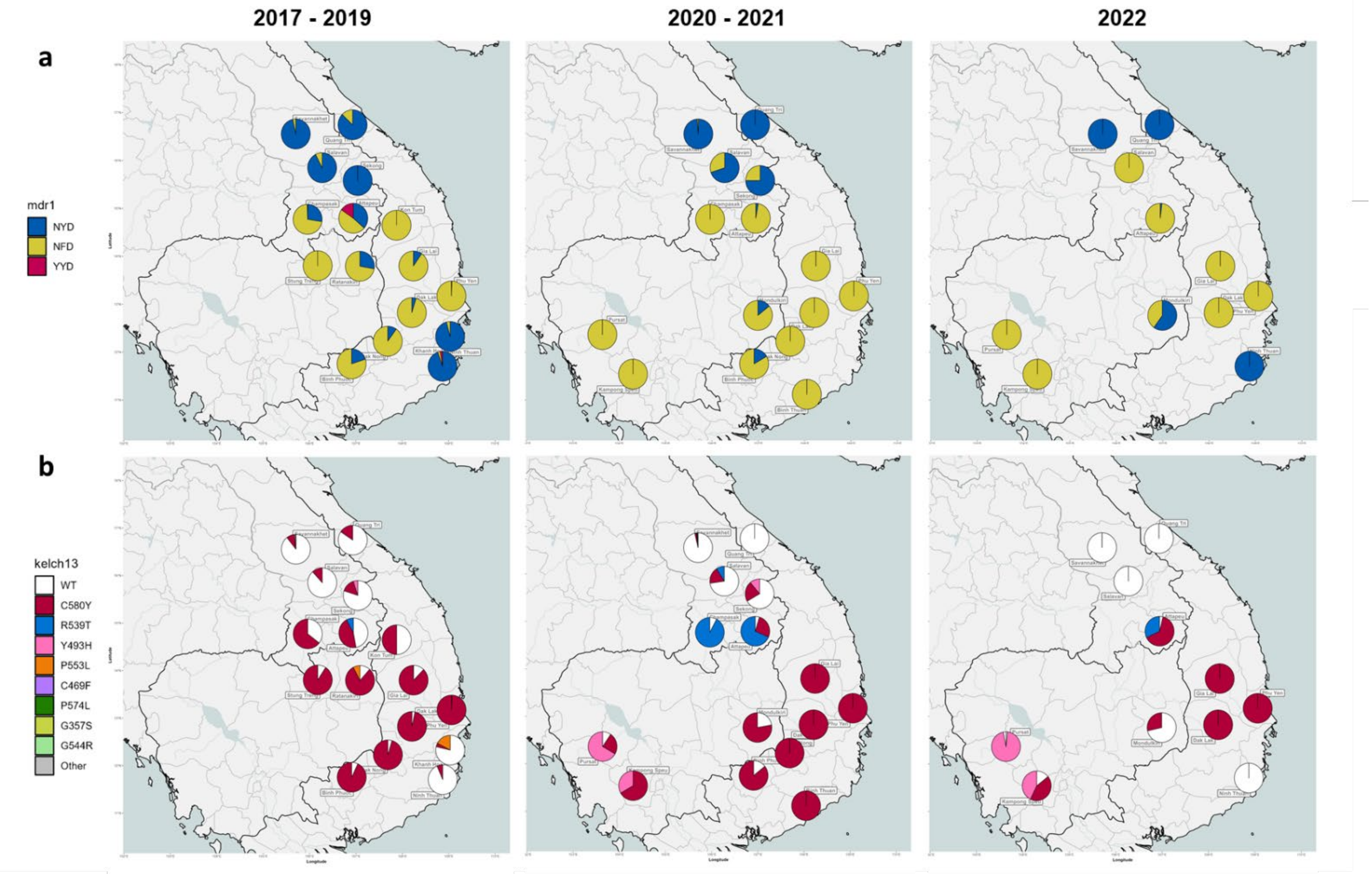
2017 - 2019
2020 - 2021
2022
a
b
mdr1
NYD
NFD
YYD
kelch13
WT
C580Y
R539T
Y493H
P553L
C469F
P574L
G357S
G544R
Other

Supplementary Methods

Sample genotyping. DNA was extracted from DBS samples and processed using the SpotMalaria v2 amplicon sequencing and genotyping platform. Detailed sample processing procedures, and the set of amplicons and variants targetted, are given in the "SPOTmalaria Technical Notes and Methods". Amplicon primer sequences are available in "Supplementary File 1". Both files are available at https://www.malariagen.net/resource/29/, released as supplementary material for Jacob CG *et al.*, 2021.[14] The FASTQ files produced by sequencing were then processed by an informatics pipeline that genotyped the samples and produced the Genetic Report Cards datasets that were used in the analyses. The pipeline is available open-source at https://github.com/genomic-surveillance/AmpRecon with documentation, including a complete workflow. Here, we give an overview of functional blocks that are relevant to the results presented.

Alignment and Read Counts. Illumina short read pairs were aligned against reference amplicon sequences, obtained from the 3D7 reference genome V3; reference files are available from https://github.com/genomic-surveillance/AmpReconResources/tree/main/plasmodium/falciparum. Alignments were processed to remove reads that did not meet quality criteria, as detailed in the "SPOTmalaria Technical Notes and Methods", and `bcftools mpileup` (https://samtools.github.io/bcftools/bcftools.html) was used to extract VCF files containing the read depth of each allele at every position of interest.

Variant Genotyping. At every position of interest, nucleotide genotypes were established by analyzing allele read depths. If the position was covered by fewer than 10 reads, the genotype was considered "missing" (i.e. undetermined due to insufficient coverage). Otherwise, the sample was deemed as carrying all alleles that were in no less than 5 reads AND in at least 10% of the total reads at that position. If a single nucleotide allele met these criteria, the sample was genotyped as homozygous for that allele; if more than one allele met the criteria, the sample was genotyped as heterozygous. Amino acid alleles were genotyped by concatenating the nucleotide alleles of the adjacent positions in the codon, and then translating the codon to an amino acid. The amino acid genotype was deemed missing if any of the codon's nucleotide genotypes were missing.

Barcodes. For each sample, a 101-SNP barcode was called, simply by concatenating the genotypes from each of the positions. Barcode missingness was estimated as the proportion of sites that had a missing genotype; barcode heterozygosity was estimated as the proportion of non-missing positions that carried a heterozygous genotype. Barcode missingness was used as a quality measure, as it increases when low levels of DNA concentration reduce sequencing yield.

***kelch13* genotyping**. Nucleotide positions covering *kelch13* amino acid positions 340-695 were genotyped for each sample, using 6 overlapping amplicons. The genotypes were then scanned to detect changes in the amino acid sequence. If more than 25% of positions was missing, the *kelch13* genotype was deemed "missing". Otherwise, the sample was genotyped as "wild type" ("WT") if all the amino acid positions were called with the same allele as the 3D7 reference. If a single amino acid change caused by a homozygous nucleotide allele was detected, the sample was assigned the relevant mutation. However, if the nucleotide allele that caused the amino acid change was heterozygous, the position was considered heterozygous (i.e. harbouring both mutant and wild type parasites). Similarly, the detection of multiple amino acid changes produced a heterozygous allele.

***plasmepsin 2/3* amplifications**. *Plasmepsin 2/3* (*pm23*) amplifications were determined by combining results from two methods: breakpoint sequence identification[14,12] and qPCR.[15] Copy numbers were not quantified, so the test outcomes were only "wild type" ("WT") if there was a single copy of the gene, or "Amplified" if multiple copies were detected. When both methods produced matching results, or if only one method produced a result, that outcome was assigned as the result. In the presence of discrepancies between the two methods, a "missing" (undetermined) status was assigned.

***mdr1* amplifications**. Amplifications of the *Pf* genes *multidrug resistance 1 transporter* (*mdr1*) was detected by qPCR.[15] Copy numbers were not quantified, so the test outcomes were only "wild type" ("WT") if there was a single copy of the gene, or "Amplified" if multiple copies were detected. A "missing" (undetermined) value was assigned when qPCR could not determine an amplification status.

Phenotype Predictions. The amino acid genotypes at specific positions were used to derive predictions of the drug resistance status for a variety of drugs. The prediction rules are detailed in "Phenotype Rules", available at http://ngs.sanger.ac.uk/production/malaria/Resource/29/20200705-GenRe-05-PhenotypeRules-0.39.pdf, released as supplementary material for Jacob CG et al., 2021.[14]

References

1 Global Malaria Programme (GMP). Report on antimalarial drug efficacy, resistance and response: 10 years of surveillance (2010-2019). (World Health Organisation, 2020).

2 World Health Organizaion. Mekong malaria elimination programme: epidemiology summary, volume 4, October-December 2018. (2018).

3 World Health Organizaion. Mekong malaria elimination programme: epidemiology summary, volume 12, October-December 2020. (World Health Organisation 2020).

4 World Health Organizaion. Mekong malaria elimination programme: epidemiology summary, volume 20, October–December 2022. (World Health Organisation 2023).

5 World Health Organizaion. Mekong malaria elimination programme: Accelerating malaria elimination in the Greater Mekong, bulletin 10. (2022).

6 National Centre for Parasitology, E. a. M. C. C. Kingdom of Cambodia National Malaria Programme Review 2019. (2019).

7 Srean, C. *et al.* Descriptive assessment of COVID-19 responses and lessons learnt in Cambodia, January 2020 to June 2022. *BMJ Global Health* **8**, e011885 (2023). https://doi.org/10.1136/bmjgh-2023-011885

8 World Health Organization. Lao PDR Coronavirus Disease 2019 (COVID-19) Situation Report #12. (2020).

9 A3M Global Monitoring GmbH.

10 Vietnam Ministry of Health. National Guideline of Malaria Diagnosis and Treatment. (Hanoi, 2020).

11 Cao, V. A. T. *et al.* Exploring the feasibility of introducing triple artemisinin-based combination therapy in the malaria treatment policy in Vietnam. *Malaria Journal* **22**, 326 (2023). https://doi.org/10.1186/s12936-023-04763-4

12 Wikipedia contributors. Vietnamese government response to the COVID-19 pandemic. (2025). <https://en.wikipedia.org/w/index.php?title=Vietnamese_government_response_to_the_COVID-19_pandemic&oldid=1271927022>.

13 Wikipedia. *Vietnamese government response to the COVID-19 pandemic*.

14 Jacob, C. G. *et al.* Genetic surveillance in the Greater Mekong subregion and South Asia to support malaria control and elimination. *Elife* **10** (2021). https://doi.org/10.7554/eLife.62997

15 Ansbro, M. R. *et al.* Development of copy number assays for detection and surveillance of piperaquine resistance associated plasmepsin 2/3 copy number variation in Plasmodium falciparum. *Malaria Journal* **19**, 181 (2020). https://doi.org/10.1186/s12936-020-03249-x
